# Supplementary material for: Molecular Genetic Basis of Reproductive Fitness in Tibetan Sheep on the Qinghai-Tibet Plateau
Source: Genes (Basel). 2025 Jul 29;16(8):909. doi: 10.3390/genes16080909 (PMC12385295; doi:10.3390/genes16080909)
Supplement: Supplementary file 1 [file genes-16-00909-s001.zip › Supplementary Figures.pdf]

Molecular Genetic Basis of Reproductive Fitness in Tibetan Sheep on the Qinghai-Tibet Plateau

Wangshan Zheng, Siyu Ge, Zehui Zhang, Ying Li, Yuxing Li, Yan Leng, Yiming Wang, Xiaohu Kang, Xinrong Wang

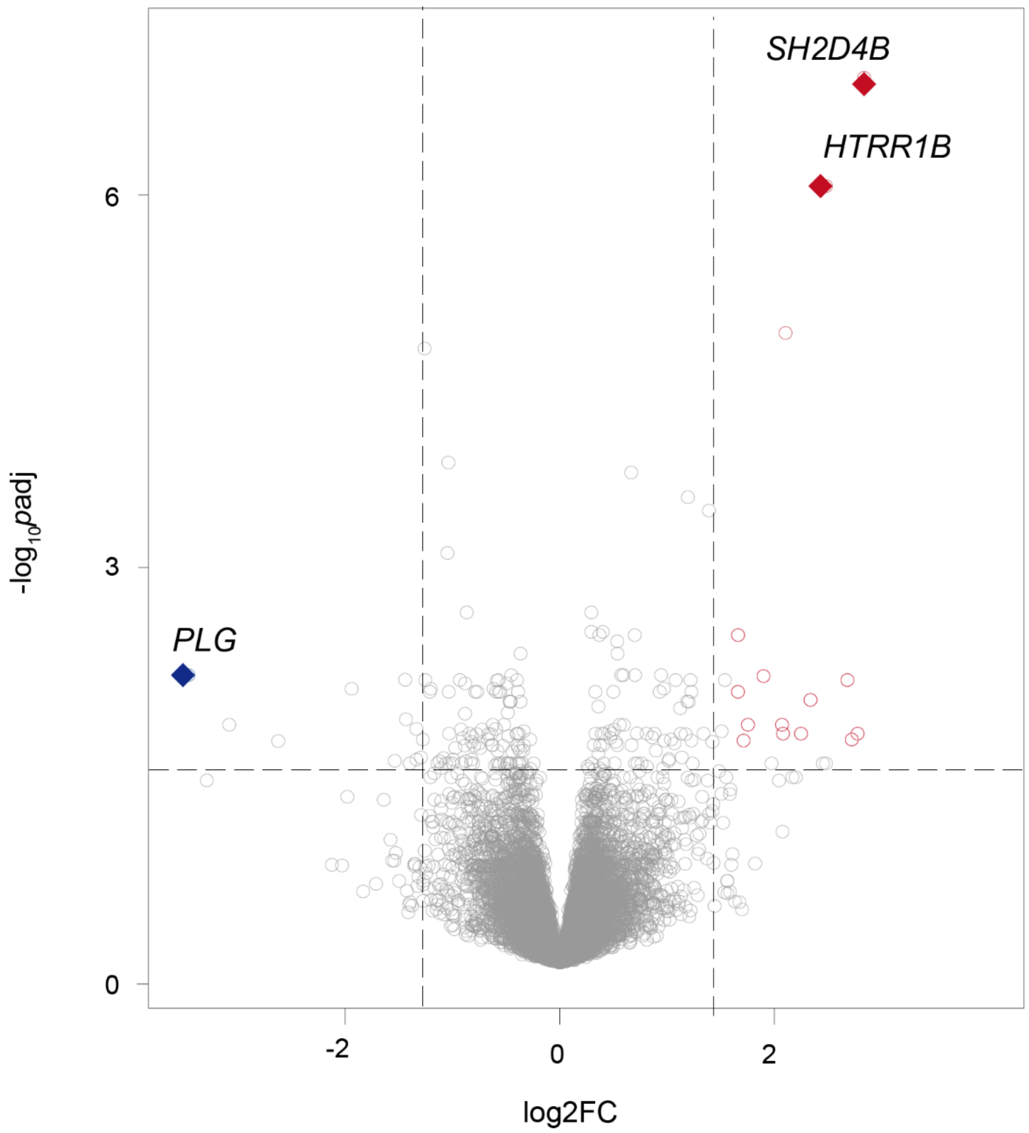

Supplementary Figure S1 Differential expression analysis between Tibetan and Hu sheep, identifying 235 THDEGs (labeled genes represent the top three most significant hits by $\log_2$FC).

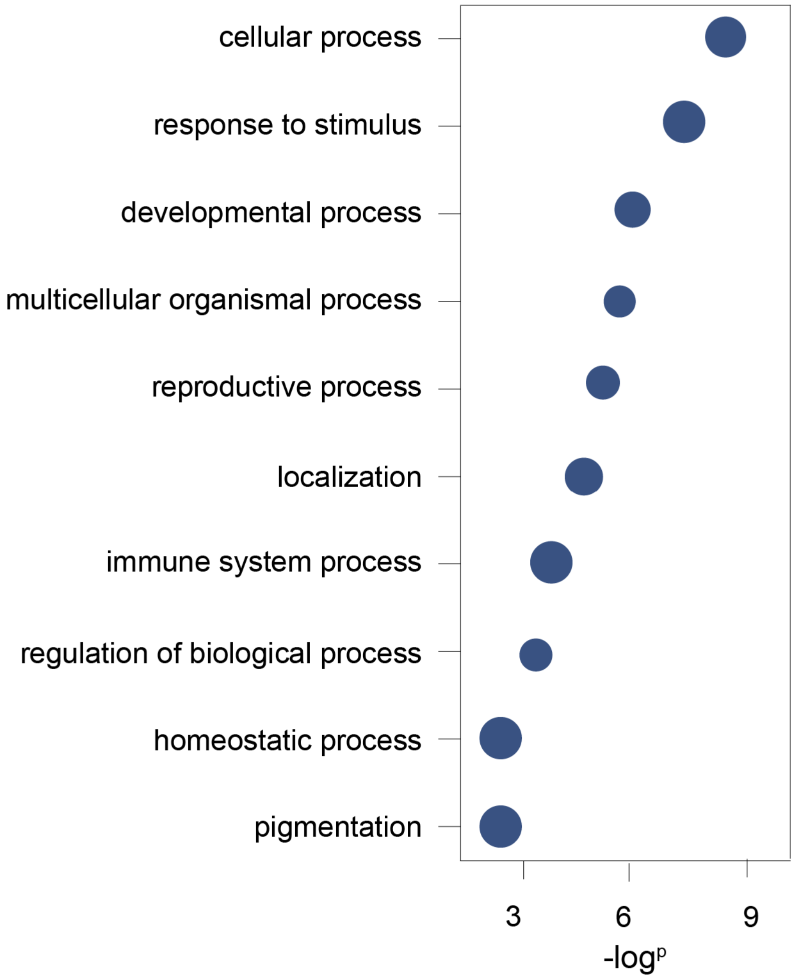

Supplementary Figure S2 THDEGs exhibited distinct enrichment profiles associated with cellular homeostasis, developmental regulation, stress response, and reproductive processes. KEGG/GO enrichment was generated by Metascape (https://metascape.org).(Zhou, et al. 2019)

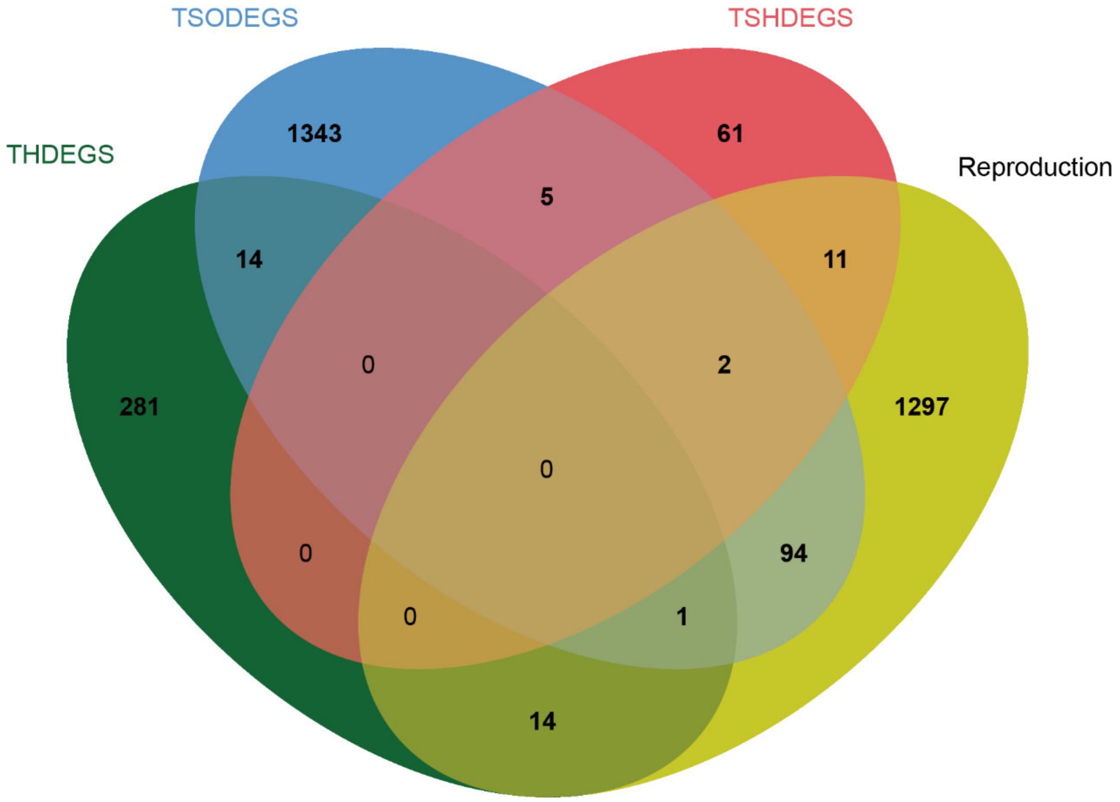

Supplementary Figure S3 112 overlapping DEGs (13 THDEGs, 5 TSHDEGs, and 94 THODEGs). Venn diagrams show the number of intersection genes among THDEGs, TSHDEGs, THODEGs and reproductive pathway genes. Venn diagrams are implemented in ggplote2 (R4.1.4).

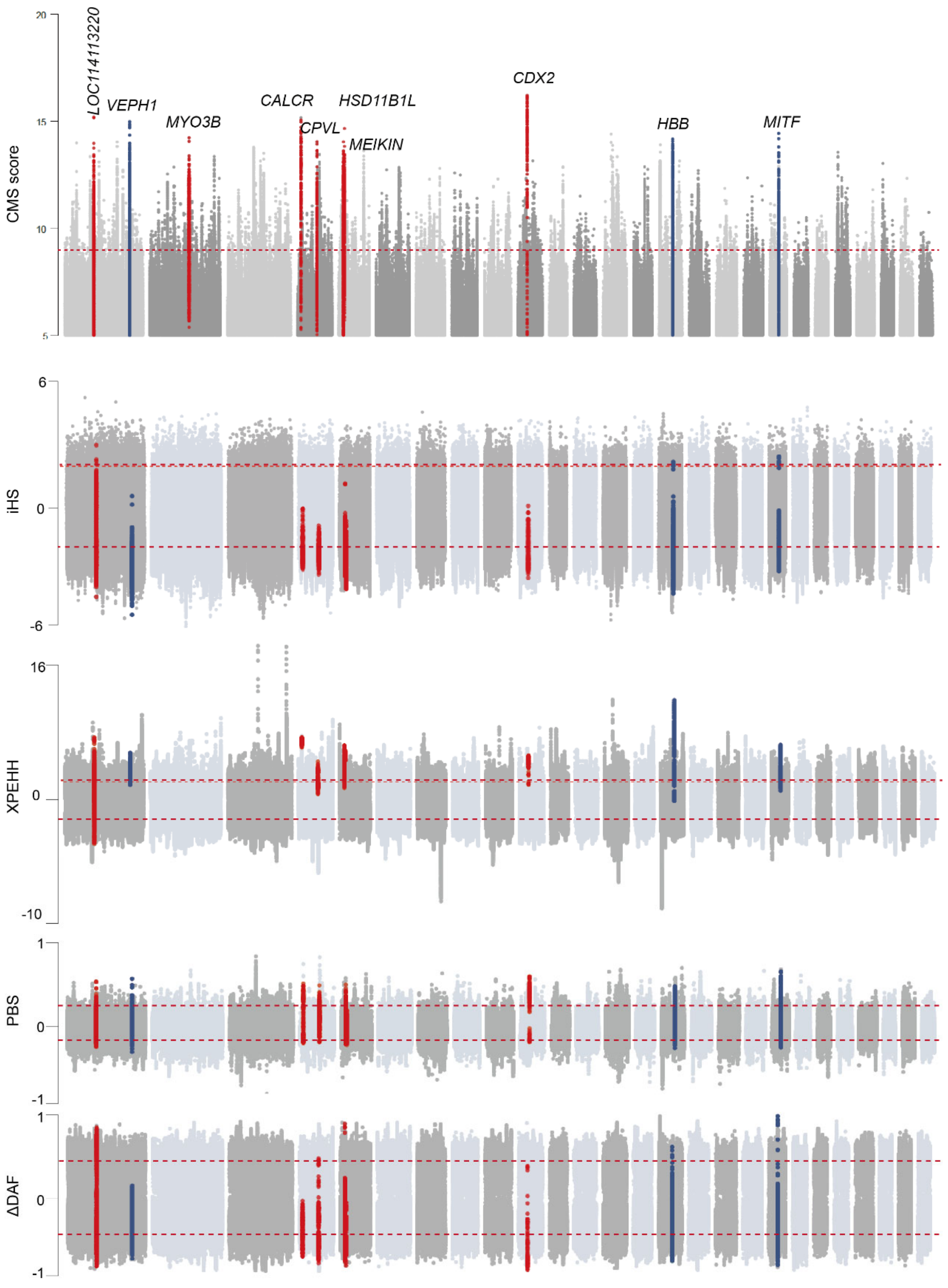

Supplementary Figure S4. Genome-wide selection scan in Tibetan sheep. We employed four selective sweep metrics (XPEHH, iHS, PBS, and ΔDAF) as input for Composite Measure of Selection (CMS). Genes in the top 10 CMS windows are annotated with gene symbols. All four metrics demonstrate convergent, statistically significant selection signals across multiple genomic regions.

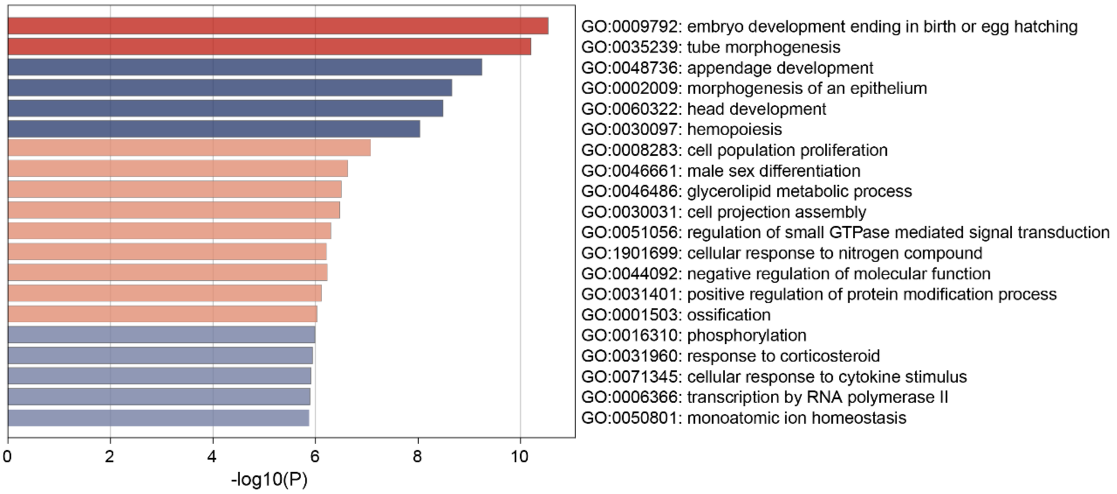

Supplementary Figure S5. Functional enrichment of Tibetan sheep TSNSGs. TSNSGs were mapped to orthologous human and mouse genes for functional enrichment analysis using Metascape(Zhou, et al. 2019). Significant enrichments (FDR<0.05) were observed for: Embryonic development ending in birth or egg hatching (GO:0009792);Tube morphogenesis (GO:0035239),Results align with transcriptome-derived GO terms from RNA-seq data.

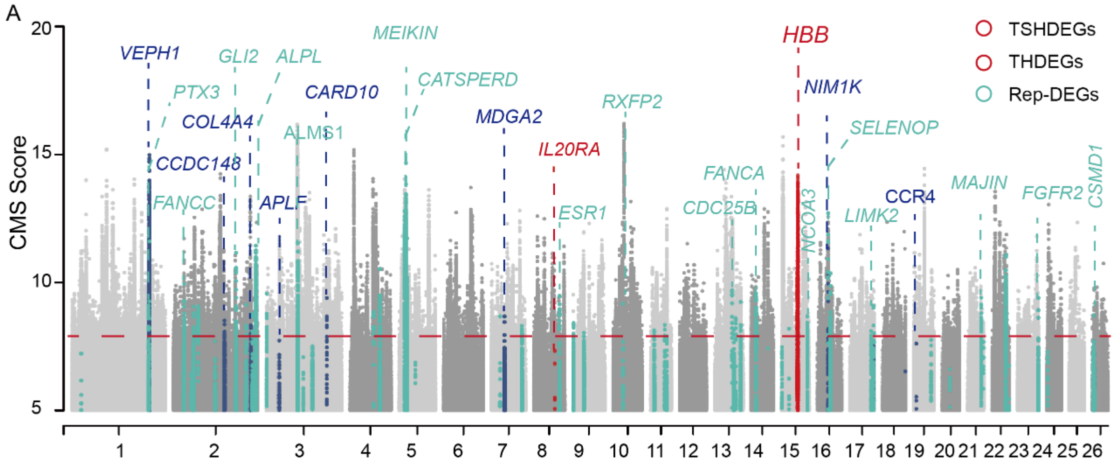

Supplementary Figure S6 Manhattan plots of selection signals in Tibetan sheep DEG sets. Genome-wide distribution of positive selection signals for:1) Tibetan sheep-vs-Small-tailed Han differentially expressed genes (TSHDEGs) and Tibetan sheep-vs-Hu DEGs (THDEGs). Rep-DEGs: Reproductive pathway DEGs (data from PathCards).

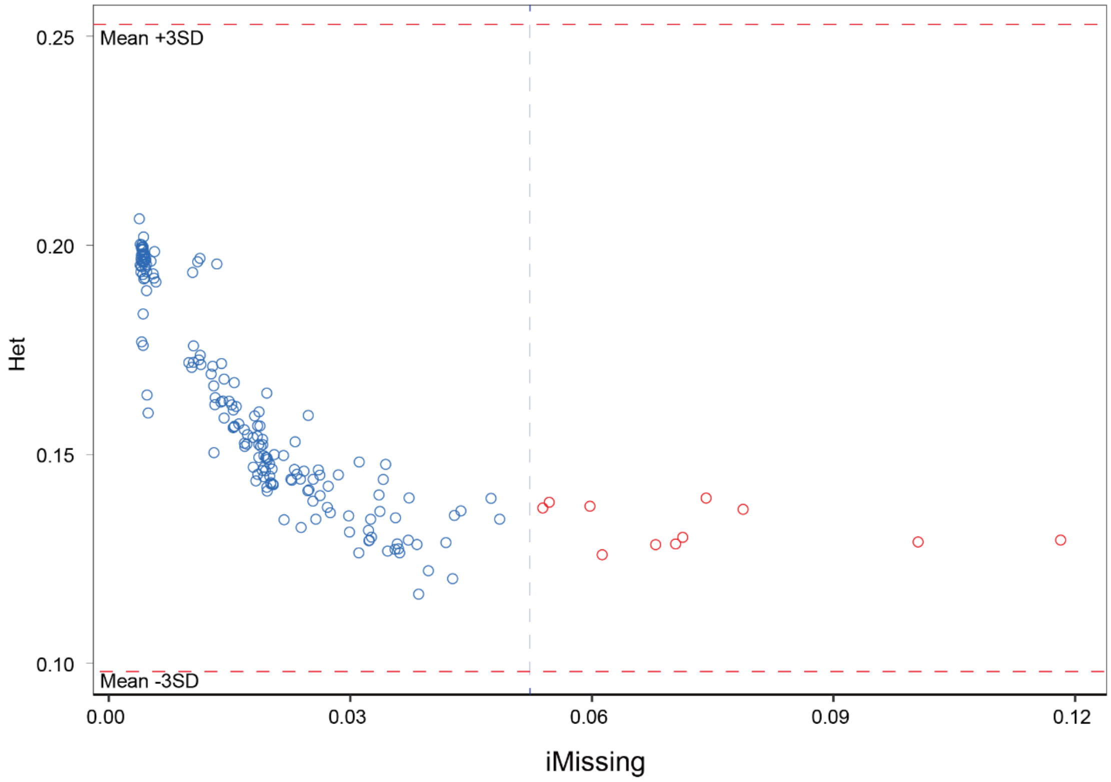

Supplementary Figure S7. Individual-level quality control of whole-genome data. Individuals exhibiting: Missing genotype rate ⩾ 3%; Heterozygosity beyond mean ± 3 SD;were excluded (*n=11 samples removed*). Thresholds indicated by red dashed lines**.**

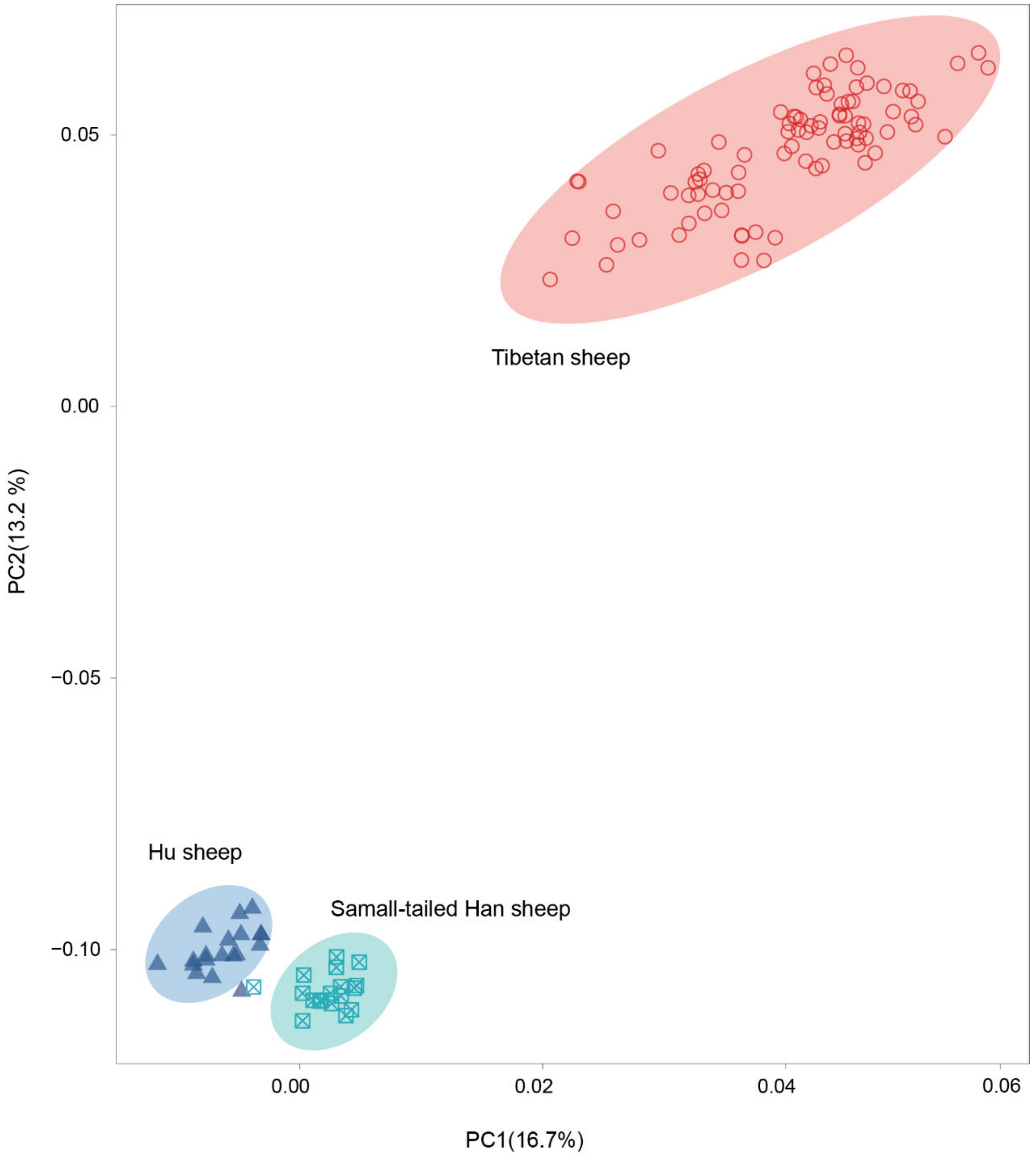

Supplementary Figure S8. Principal component analysis (PCA) of sheep populations. Clear separation among breeds along PC1/PC2 axes: Tibetan sheep, Hu sheep, Small-tailed Han sheep. Two potential hybrid individuals showing admixture were removed from subsequent analyses.

Zhou Y, Zhou B, Pache L, Chang M, Khodabakhshi AH, Tanaseichuk O, Benner C, Chanda SK. 2019. Metascape provides a biologist-oriented resource for the analysis of systems-level datasets. Nat Commun 10:1523.
